# Shedding light: an evidence-based approach to adapting cognitive bias tests for reptiles

**DOI:** 10.1098/rsbl.2026.0318

**Published:** 2026-10-28

**Authors:** Jessica T. Turner, Eduardo J. Fernandez, Alexandra L. Whittaker

**Affiliations:** The Adelaide University, Adelaide, South Australia, Australia

**Keywords:** attention bias, judgement bias, affect, animal welfare

## Abstract

Quantifying animals' subjective experiences, or affective states, has become an increasing focus within welfare science. Physiological measures can reliably detect emotional arousal, but assessing valence, whether an experience is positive or negative, remains challenging. Behavioural indicators can assist in this regard, yet they often require species-specific interpretation and may introduce observer bias. These difficulties are compounded when working with relatively understudied taxa such as reptiles. Cognitive bias tests, particularly attention and judgement bias paradigms, provide a promising approach for assessing affect. Attention bias tests are rapid, cost-effective and require minimal training, whereas judgement bias tests, although more time-intensive, can detect subtle shifts in affect and can be repeated over time to monitor long-term changes. Despite their potential, few studies have applied these methods to reptiles, and necessary species-specific adaptations remain largely untested. Pilot studies are therefore essential to establish learning rates, identify effective cues and determine motivational preferences across reptile species. Developing a clearer link between cognitive bias and welfare outcomes will also be important. Given the increasing number of reptiles housed in private and professional settings, there is a pressing need for robust empirical tools to assess their welfare. Cognitive bias testing represents a promising and underused method that could significantly advance welfare science in reptiles.

## 1. Introduction

Public interest in the quality of life, or welfare, of animals has increased substantially over recent decades [1–4]. Similarly, more widespread recognition of animal sentience, both within law and everyday life, has shifted the way we assign the value of animals, not just in what they can provide to humans as a resource, but as beings with inherent value and rights to a good quality of life [5,6]. This shift has arguably driven the expansion of animal welfare science. Scholars in this discipline typically use measures of behaviour and/or physiology with the aim of inferring animal welfare state [7]. Much of the development of welfare science and assessment has been guided by the Five Freedoms. The Five Freedoms emphasizes the avoidance of negative states but failed to promote the presence of positive experiences in animals as a fundamental component of welfare [8–10]. As a result, more recent assessment tools such as the Five Domains have shifted focus towards assessment of the full range of an animal's subjective experiences, including when ‘good’ welfare has been achieved [10–12]. Good welfare is often brought about by the presence of positive stimuli, such as enrichment, and by providing opportunities for choice and control which are evidenced through behavioural indicators such as active use of presented alternatives [13]. Although one taxon is consistently overlooked in welfare research: reptiles.

Reptiles are kept in captivity for companionship, conservation, education and research. While reptile pet ownership rates are often lower than for other pets, the population can still include millions of animals (e.g. 2.9% and 2.7% in the UK and Australia, respectively), with populations in UK zoos increasing by 47% from 2003 to 2023 [14–16]. This trend highlights the need for accessible, evidence-based welfare assessment tools for reptiles. Reptiles have been under-represented in welfare science, potentially due to early assumptions that reptiles were behaviourally and cognitively limited [17–20]. Yet, research has highlighted that reptiles possess diverse behaviours and cognitive abilities, including spatial learning and memory [21,22], and therefore have the capacity to experience the full range of the welfare spectrum from poor to good [23].

While it is now widely acknowledged that animal carers should be striving to create a state of positive welfare in the animals they care for, it can be challenging to assess when that state has been achieved as there are less well-developed measures of positive welfare overall. This may be especially so for reptiles, due to less characterization of positive measures of affect. Work to develop methods to better characterize the range of welfare states experienced by animals is important for facilities that care for large numbers of animals who may be time- or resource-poor, as knowing which changes will lead to the greatest benefit can help guide a targeted approach to future improvements. Typically, welfare scientists choose to measure long-term welfare state through characterizing short-term affective states of animals across a relevant period. Animals' affective states are typically conceptualized along the dimensions of arousal (high vs low) and valence (positive vs negative) [24]. Positive states may include those arising from bonding and communication in social species. Negative states may result from behavioural restriction such as isolation or limited environmental complexity, and are often evidenced by stereotypic or self-injurious behaviours [25–28]. Yet, challenges abound. Many existing affect measures are sound at detecting short-term changes but may be too simplistic to fully capture long-term affective states [29,30]. In reptiles, this issue is compounded by the lack of baseline data on natural behaviour and physiological ranges, making it challenging to identify the nature or valence of reptile emotional responses [18]. While there are some well-characterized behaviours associated with reptile stress, comfort behaviours (or those of positive affect) are often less obvious and more ambiguous [31,32]. Currently used measures for reptile welfare assessment include activity budgets, behavioural diversity indices, responses to novelty and physiological sampling [18,33–39], but there is a need for further development of welfare assessment tools that better elucidate both strength and valence of reptile affect. Cognitive bias tests may be one such novel assessment tool.

Cognitive bias tests offer a promising new welfare measure for reptiles as they can measure not only the valence (and sometimes arousal) of affect but can also provide insight into the longer-term affective states. Additionally, because they use multiple behavioural measures within the test, and commonly measure clearly defined parameters, they are arguably less subjective than other measures which could be susceptible to observer bias. Despite its promise, cognitive bias has been understudied in reptiles, with only recent emerging interest as discussed below.

## 2. Cognitive bias as a welfare measure

Cognitive bias refers to the influence of affective state on cognitive processing and decision-making [24]. Positive or negative past experiences can bias responses to ambiguous stimuli. An ambiguous stimulus is defined as one which has not been encountered before but has similar qualities to previously encountered stimuli that may have been positive or negative in nature. For example, a lizard may hear a predatory bird call before being attacked and escaping. When exposed to a non-predatory bird call, they may recall their previous experience and decide whether this new call alerts them to an imminent attack or not. Since deciding whether or not to take shelter or continue with regular activities could impact a lizard's survival in the wild, cognitive bias may therefore offer evolutionary advantages. Cognitive bias tests, including judgement bias tests (JBT) and attention bias tests (ABT), have been widely used in human research and increasingly applied to animals [40–42]. These measures can also detect long-term mood shifts following environmental changes [43–46]. In the following section, we consider two types of cognitive bias tests, attention and judgement bias, and their potential application to reptiles.

### (a) Attention bias tests

The theory of attention bias states that animals in long-term negative affective states show greater attention to negative stimuli than animals experiencing positive affective states [41]. So, while all animals will be attentive to potential threats, the speed at which the animals resume regular activity can vary. Attention bias explains this discrepancy by attributing these differences to the individual's affective state [47,48]. ABT involve exposing the animal to a threatening or distracting stimulus, then measuring various responses, such as attention towards the threat, latency and other behavioural measures. Latency is commonly described as the time taken to complete a task, such as moving to a specified point, or reaching a food bowl and eating [49]; however, in ABT it is the time taken to resume regular behaviours in the presence or absence of a threat.

Attention bias is not as well researched as judgement bias in animals; therefore, methodologies are still being developed and tested. As attention bias tests have been a relatively recent development in animal science, some argue that the impact of arousal on the test requires investigation, and the test's ability to identify positive valence—in opposition to negative valence also requires further validation [1].

### (b) Judgement bias tests

Judgement bias tasks identify an animal's affective state by assessing the judgement, either a positive or negative judgement, made when confronted with an ambiguous stimulus. Characteristics of the animal's response, such as freezing or latency to approach, are used to determine if the judgement made is in enhanced expectation of a positive or negative event [50]. The terms positive and negative are used in this paper to communicate the concept of opposing states. In this test, the animal is trained to discriminate between two stimuli which evoke a set of behavioural responses. When the animal approaches one stimulus, a reward is obtained, often that of palatable food. The other stimulus is either non-reinforced or reinforced with a negative stimulus (e.g. unpalatable food) or a less preferred reward, to produce either a neutral or avoidance response. When presented with an ambiguous stimulus of the same type (i.e. visual) but intermediate between the trained positive and negative stimulus, the animal's long-term affective state impacts their response. If the animal avoids this ambiguous stimulus, or delays their approach, they are most likely experiencing a negative long-term affective state. Emerging evidence has also linked anticipatory behaviour with test outcomes. Anticipatory behaviour is associated with the anticipated acquisition of a reward. When anticipatory behaviours are recorded in conjunction with a judgement bias test, higher or excessive levels of appetitive/anticipatory behaviour are correlated with poorer welfare [51]. Additionally, if the ambiguous cue is approached, or approached quickly, they are more likely to be experiencing positive long-term affect. There are multiple ways to present the ambiguous cue. As described above, a single ambiguous cue can be used, which is central between the positive conditioned stimulus (+CS) and the negative conditioned stimulus (−CS) [52]. Alternatively, three or more ambiguous cues can be used to assess responses in finer detail [53]. JBT can either be an active choice task or a Go/No-Go task (summarized in table 1).

**Table 1.** Summary of attention and judgement bias tasks. Conditioned stimulus is identified as CS and can be positive or negative; centre is C, near positive is NP, near negative is NN.

bias type	task	reinforcement	training	ambiguous cue	sensitivity	measures	expected outcome	multiple trials
attention	looking time	N/A	not required	N/A	N/A	ethogram; behavioural index; difference in latency; looking time	prolonged attention to threat—poorer affect	no*
judgement	Go/No-Go	+CS/−CS e.g. palatable and unpalatable food	required	yes	C or NP, C, NN	ethogram; behavioural index; choice; latency; JBT index	approaches ambiguous cue quickly—positive affect slow or no approach—poorer affect	yes
judgement	active choice	++CS/+CS e.g. high priority food and a lesser priority food	required	yes	C or NP, C, NN	ethogram; behavioural index; choice; latency; JBT index	performs action quickly when presented with ambiguous cue—positive affect slow or no action—poorer affect	yes

* See discussion in §2.9.

In an active choice task with positive reinforcement, two +CS are used, generally a high priority appetitive (++CS) and a lesser priority appetitive (+CS) [50]. This approach is considered to be less aversive and ethically more acceptable in a test that is often repeated over a few months or more. In this design, it is expected that when presented with an ambiguous stimulus, the animal will approach; however, the latency to approach should be slower when anticipating a low-priority reward than a high-priority one. The speed of approach towards the ambiguous stimulus is used to indicate the animal's affective state; that is, if food was the reinforcer, a fast approach would be considered an indicator of a positive affective state, over a slower one. Active choice tasks can also be designed with negative reinforcement. In these designs, the animals are trained to take action to receive a reward and to avoid a negative stimulus. When presented with an ambiguous cue, the type of action they take indicates if they are expecting a reward or punishment [54]. Bethell's [50] review discusses this design further with examples; however, as it is less suited to assessing welfare, it will not be discussed further in this review.

The alternative option is a Go/No-Go when a +CS and a −CS are used [50]. This encourages the animal to ‘go’ when anticipating a reward and ‘not go’ if anticipating a neutral or mildly aversive stimulus. Eliciting the ‘go’ response would be considered an indicator of positive affect, with a faster response indicating the level of arousal. The ‘no-go’ response may indicate a neutral to negative affective state, with the absence or presence of avoidance behaviour providing an indication of arousal in relation to the negative affect. Avoidant behaviour may include attempts to hide, freeze and escape.

While there may be benefits, there are also many critiques of JBT. Some critiques include that the training involved is time-intensive [55] and the activity levels of animals may impact results, particularly when latency is the main measure [42]. Additionally, concerns have been raised that with repeated testing the ambiguous cue may lose ambiguity [56], meaning behavioural extinction occurs. The training involved and the test itself may also constitute cognitive enrichment and impact results [55]. Interpretation can also be an issue, with some studies reporting the opposite results to the expected outcome [1,57]. Further research is needed to investigate these issues further and develop contingencies to overcome them.

### (c) Application to reptiles

Both ABT and JBT are minimally invasive and are probably suitable for reptiles. However, only three studies have explored cognitive bias in reptiles [50,52,53], leaving major gaps in method development and validation. This review integrates current knowledge from reptile behavioural research with broader cognitive bias literature to inform the adaptation of ABT and JBT paradigms for reptiles. It discusses methodological considerations from piloting through to test design, outputs and statistical analysis.

## 3. Methodological considerations for reptiles

Adapting cognitive bias tests for reptiles requires close attention to biological, behavioural and environmental factors that differ from commonly studied taxa. This section outlines the principal considerations for successful adaptation, focusing on life history and physiology, behavioural ecology and stress responses, the choice between home and arena testing environments, motivation and reinforcement, preference testing and cue selection, individual differences, sample size, data analysis and measurement strategy (see table 2 for key points and resources).

**Table 2.** Potential stimuli and test cues for reptiles. Appetitive stimuli could be used as high preference items or as reinforcers during training. Aversive stimuli could be used to promote an avoidance response or paired with a cue example to produce a trained avoidance response. Cue type and example refer to the type of stimuli that could be used during a cognitive bias test.

reptile	appetitive/positive stimuli	aversive/negative stimuli	cue type	cue example	reference
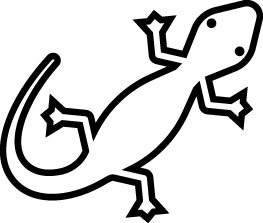 lizard	food; UV light; heat	simulated predatory attack e.g. bird puppet; predator scents; touch unpalatable food: e.g. treated food; naturally unpalatable foods	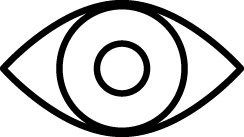 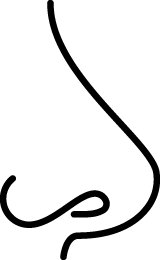	colours (e.g. grey or blue) geometric patterns bird puppet cat urine scent	[58–88]
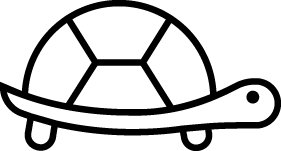 tortoise/turtle	food; UV light; heat	scents of unknown conspecific	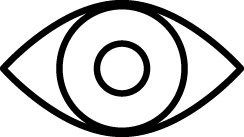	colours (e.g. yellow or red) quantities (e.g. 1 square or 10 squares)	[63,64, 72–76,80,81,83,89]
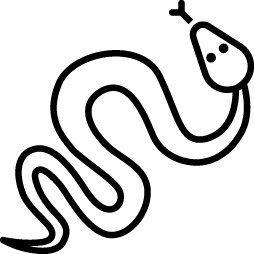 snake	food; UV light; heat		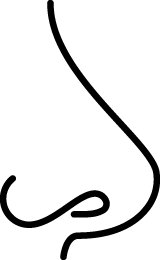 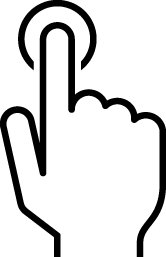	earthworm wash lemon distilled water amyl acetate artificial grass (astroturf)	[90–92] [93]
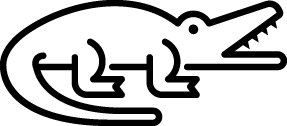 crocodilian	food; UV light; heat		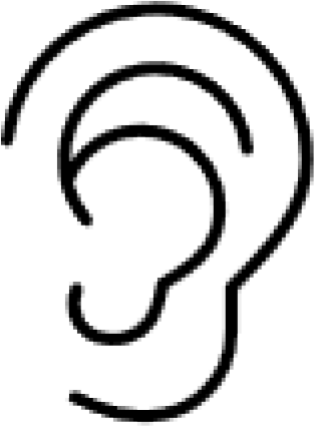	various tones	[94]

As reptiles rely on external temperatures, circadian and seasonal rhythms can influence reptile behaviour and test performance. Nocturnal species, such as the broad-headed snake, have smaller activity windows, as they balance the risk of predation with the rewards of hunting and their thermoregulatory requirements [95]. Seasonal fluctuations in temperature, even within captive environments, can alter both activity and behaviour [96]. Thermoregulation is especially important because movement speed and responsiveness vary with temperature. As a result, latency-based measures can be confounded if temperatures fluctuate during tests [97]. Therefore, the time of day and temperature of the testing area should be considered carefully depending on the target species. Developmental history also matters. Incubation temperature can affect learning speed and cognitive capacity in reptiles, making it essential to gather detailed background information on sourced animals and to control for, or include as covariates, these developmental variables in analyses [98–100]. Physical and behavioural traits that allow reptiles to thrive in various environments can also have an impact.

The ecological niche of a target species is a critical consideration in the design of behavioural tests for reptiles, as habitat specialization strongly influences how individuals interact with experimental environments. Species adapted to terrestrial, arboreal or semiaquatic habitats benefit from test arenas that reflect these ecological contexts, such as the inclusion of climbing structures for arboreal taxa or ramped access to water for semiaquatic species [101,102]. Closely linked to habitat use is foraging strategy. Ambush predators, such as the timber rattlesnake [90], may be reluctant to traverse large or exposed arenas, meaning task designs should allow responses to occur within striking distance or rely on the display of anticipatory behaviours that do not require extensive locomotion. Predator–prey status further shapes how reptiles respond to test conditions, particularly in relation to perceived risk and stress.

Many temperate-zone reptiles spend large amounts of time within a refuge [103]. Refuges are selected based on thermoregulatory properties and predator avoidance [104]. Therefore, being exposed to an open, well-lit arena with no refuge for animals may cause discomfort and heightened vigilance. Such responses can confound behavioural outcomes but may be mitigated through gradual habituation, the provision of refuge options, or by conducting tests within the home enclosure. Reptiles vary widely in their antipredator strategies. For example, the southeastern *Morethia* skink displays behavioural responses to a range of simulated threats including visual predator models, predator vocalizations, tactile stimulation and predator odours [86,105–107].

Human presence is another important factor affecting the behaviour of reptiles. Direct eye contact can elicit vigilance or escape responses in the Indian rock lizard, making it advisable to minimize overt observation and rely instead on camera systems. If moving components are involved, automation may be necessary [108]. Even when animals are habituated to observers, residual effects may persist, strengthening the case for video recording both to improve measurement accuracy and to facilitate inter observer reliability assessment. Additionally, emerging evidence suggests reptiles can alter behaviour depending on familiarity with a human handler and in the context of handling [109]. A new automated caging and testing solution ‘ReptiLearn’ has demonstrated the capacity for bearded dragons to live in an automated system and learn a spatial task without human interaction [110]. This could eliminate the variability that may arise between human observers and handlers. This advancement highlights how emerging technologies can improve the rigour of reptile behavioural and cognitive research. Finally, acoustic communication between conspecifics requires caution. Distress calls can trigger avoidance behaviours in weeping lizards, so ideally animals should be housed outside the visual and auditory range of the testing arena during trials [111].

Motivation is another factor that can confound results. Feeding ecology and schedules have direct implications for motivation and influence the decision around whether food is an appropriate reinforcer or stimulus. Many reptiles feed infrequently, which can depress food-seeking behaviour during training and testing [112]. Other than feeding frequency, the size of the food offered is another important factor. Snakes often reject small quantities of food, while smaller reptiles or species that feed sporadically can become satiated quickly, reducing the number of feasible trials per session [93,113]. Non-food incentives that are biologically salient may include access to heat, ultraviolet light or conspecifics during breeding periods. Burghardt describes heat being used successfully as a reward as early as 1971, wherein lizards were trained to depress a lever to switch on an overhead heat source [20,114]. This demonstrates the potential for heat to be used as a reinforcer, although it appears to be unexplored in modern studies. Preliminary work should identify high-value reinforcers empirically to avoid assumptions that may not hold for the target species.

### (a) Cognitive bias protocol considerations for reptiles

#### (i) Species and Individual differences

Preference testing is essential to identify both appetitive and aversive stimuli because human judgements about animal preferences are often unreliable, even among experienced caretakers [115,116]. For example, newly hatched domestic chicks preferentially pecked green targets and avoided red ones, suggesting that red could serve as a suitable aversive visual cue in an attention bias context [117]. Reptiles, like other taxa, may exhibit side biases or innate colour preferences, which can influence test performance and interpretation [65,117–121]. These biases, when identified, can be addressed by counterbalancing stimulus positions or, where appropriate, removing problematic features such as particular colours.

Temperament, defined as consistent individual differences in behaviour across time, is increasingly recognized in reptiles and can shape antipredator responses and test engagement [122]. Learning speed and task engagement vary widely among individuals and species. For instance, skinks have required anywhere from 7 to 75 trials to reach criterion in associative learning tasks, and some lizards can take an hour or more to initiate movement, which significantly prolongs sessions [66,123]. Identifying and testing assumptions made during planning, including expected training duration, trial frequency and likely reinforcers, can improve study robustness and expand species-specific knowledge. Species with rapid learning and tolerance for multiple daily trials may be suitable for judgement bias tasks, whereas species with limited learning evidence or long latencies may be better candidates for attention bias paradigms.

#### (ii) Considerations for sample size

Sample size determination in cognitive bias studies requires careful consideration of individual variability, study objectives and the desired level of statistical power. Test designs that involve learning components are prone to substantial attrition, with more than half of individuals sometimes failing to reach training criteria within short time frames [124]. Although extending training periods may increase the proportion of individuals that meet criterion, longer protocols are often impractical. Repeated measures designs can partially offset small sample sizes by increasing data per individual. However, repetition carries risks. In attention bias paradigms, repeated exposure may lead to habituation or sensitization to the stimulus, while in judgement bias tasks it can reduce ambiguity through behavioural extinction [56,125]. Judgement bias paradigms generally generate richer datasets than attention bias tests because they allow multiple ambiguous trials per individual, whereas attention bias tests are often limited to single or few exposures. This enables a wide range of analytical approaches, including averaging response latencies across sessions, recording the presence or absence of anticipatory or aversion behaviours, and calculating composite measures such as the Judgement Bias Index [126,127]. The Judgement Bias Index integrates latency to approach positive, ambiguous and neutral or negative cues into a single score ranging from 0 to 1, with values above or below a certain threshold indicating the valence of the bias (positive or negative) [126]. Task design also affects statistical sensitivity. Meta-analytic evidence suggests that active choice judgement bias tasks produce larger manipulation effect sizes than Go/No-Go designs across reinforcement types, making them particularly advantageous when sample sizes are limited [40]. This difference may partly reflect false negatives in Go/No-Go paradigms, where a non-response can occur for reasons unrelated to affective state, such as lapses in attention during cue presentation [40].

#### (iii) Considerations for data analysis

A range of analytical frameworks are available for cognitive bias data. Binary outcomes (e.g. Go/No-Go responses) can be summarized proportionally or analysed using binomial logistic regression for more detailed inference [128]. Behavioural count data may be appropriately modelled using Poisson regression [129]. When working with small sample sizes, the application of non-parametric tests, such as the Kruskal–Wallis test, may be appropriate. For paired comparisons before and after affective manipulations, the Wilcoxon signed rank test, or paired samples sign test, provide suitable alternatives. Ultimately, analytical choices should align with the structure of the data, available sample size and the assumptions underlying each statistical model.

Latency is straightforward to calculate and compare with baseline values, but it is vulnerable to confounds, including reduced motivation and temperature-dependent variation, and should be avoided as the only outcome measure [49,130]. Behavioural metrics should be defined in a species-specific ethogram with particular emphasis on anticipatory behaviours, aversive responses and exploratory actions. Attention bias tests offer practical advantages because they are simple, rapid and cost-effective, yet they are not readily repeatable; animals can quickly lose interest, and intervals of at least a week may be needed to restore responsiveness where repetition is unavoidable [125].

#### (iv) Additional considerations

In order to investigate affect through cognitive bias testing, a manipulation of the animal's affect is required. Affective states in reptiles can be manipulated through a variety of methods including environmental modifications [38,131,132], a reduction or increase in handling [133,134], and changes to social conditions [135]. Each of these approaches may produce different magnitudes of effect depending on the target species, its natural history, and its previous housing experiences. Environmental changes, such as modifying size, lighting, temperature, enclosure complexity or access to basking opportunities, have the potential to influence both arousal and valence in measurable ways [136–138]. Handling procedures can also shape affect, as the frequency, duration and intrusiveness of human interaction may either elevate stress or reduce it through habituation or training [139]. Social manipulations, particularly the presence or absence of conspecifics, can be meaningful for species in which sociality influences motivation, vigilance or comfort. Training-related manipulations, including varying reinforcement schedules or task difficulty, likewise alter affective state by creating conditions of predictability or uncertainty. The choice of manipulation should therefore be tailored to the species’ biology and to the specific welfare questions guiding the assessment. Researchers should also be mindful that husbandry conditions can limit generalizability of results, meaning that even studies with larger cohorts may best apply to conspecifics maintained under similar conditions [140,141].

## 4. Conclusions

This review provides a foundation for the future development of cognitive bias methodologies in reptiles, while also highlighting the substantial refinement required to ensure these approaches are ecologically valid, species‑appropriate and applicable across captive contexts. Explicitly identifying and articulating underlying assumptions at the planning stage is a critical first step, as this enables researchers to recognize where empirical validation is required and where methodological decisions may inadvertently constrain interpretation. Pilot studies play a central role in this process, offering essential insight into species‑specific reinforcers and aversive stimuli, training schedules, task durations and other practical design parameters that are rarely transferable across taxa.

Although no amount of planning can eliminate all uncertainty, embedding cognitive bias testing within a framework of iterative refinement and continuous improvement will progressively enhance both the reliability and interpretability of outcomes. Such an approach is particularly important for reptiles, whose behavioural expression, motivation and learning processes often differ markedly from those of the mammalian species in which cognitive bias paradigms were first developed.

Reptile welfare remains comparatively understudied despite the increasing numbers of individuals held in private ownership and public zoological collections, underscoring the need for welfare assessment tools that operate at the level of the individual and are both practical and scientifically robust. Cognitive bias testing represents a promising avenue in this regard but remains underexplored. Continued research is therefore essential, not only to expand understanding of the relationship between cognitive bias and affective state in reptiles, but also to support the development of welfare assessment tools that are grounded in reptile biology rather than adapted uncritically from mammalian models. In doing so, cognitive bias testing has the potential to make a meaningful contribution to evidence‑based reptile welfare assessment and management.

## Data Availability

This article has no additional data.
